# Cholinesterase Inhibition Activity, Alkaloid Profiling and Molecular Docking of Chilean *Rhodophiala* (Amaryllidaceae)

**DOI:** 10.3390/molecules23071532

**Published:** 2018-06-26

**Authors:** Luciana R. Tallini, Jaume Bastida, Natalie Cortes, Edison H. Osorio, Cristina Theoduloz, Guillermo Schmeda-Hirschmann

**Affiliations:** 1 Grup de Productes Naturals, Departament de Biologia, Sanitat i Medi Ambient, Facultat de Farmàcia, Universitat de Barcelona, 08028 Barcelona, Spain; lucianatallini@gmail.com (L.R.T.); jaumebastida@ub.edu (J.B.); 2 Grupo de Investigación en Sustancias Bioactivas, Facultad de Ciencias Farmacéuticas y Alimentarias, Universidad de Antioquia UdeA, Calle 70 No, 52-21, Medellín 050010, Colombia; natalie.cortes@udea.edu.co; 3 Departamento de Ciencias Básicas, Universidad Católica Luis Amigó, SISCO, Transversal 51A No. 67B-90, Medellín 050034, Colombia; edison.osorio@gmail.com; 4 Laboratorio de Cultivo Celular, Facultad de Ciencias de la Salud, Universidad de Talca, Talca 3460000, Chile; ctheodul@utalca.cl; 5 Laboratorio de Química de Productos Naturales, Instituto de Química de Recursos Naturales, Universidad de Talca, Talca 3460000, Chile; 6 Programa de Investigación de Excelencia Interdisciplinaria en Química y Bio-orgánica de Recursos Naturales (PIEI-QUIM-BIO), Universidad de Talca, Talca 3460000, Chile

**Keywords:** *Rhodophiala*, alkaloids, molecular docking, AChE, BuChE, GC-MS

## Abstract

Amaryllidaceae plants are the commercial source of galanthamine, an alkaloid approved for the clinical treatment of Alzheimer’s disease. The chemistry and bioactivity of Chilean representatives of *Rhodophiala* genus from the family of Amaryllidaceae have not been widely studied so far. Ten collections of five different Chilean *Rhodophiala* were analyzed in vitro for activity against enzymes such as acetylcholinesterase (AChE) and butyrylcholinesterase (BuChE) as well as for their alkaloid composition by GC-MS. To obtain an insight into the potential AChE and BuChE inhibitory activity of the alkaloids identified in the most active samples, docking experiments were carried out. Although galanthamine was found neither in aerial parts nor in bulbs of *R. splendens*, these plant materials were the most active inhibitors of AChE (IC_50_: 5.78 and 3.62 μg/mL, respectively) and BuChE (IC_50_: 16.26 and 14.37 μg/mL, respectively). Some 37 known alkaloids and 40 still unidentified compounds were detected in the samples, suggesting high potential in the Chilean Amaryllidaceae plants as sources of both novel bioactive agents and new alkaloids.

## 1. Introduction

The vast structural and chemical diversity of natural products gives them a significant role in drug discovery [1]. Alkaloids are of particular interest in biomedicine and drug discovery research [2] due to their structural diversity and specific biological potential [3]. The Amaryllidaceae is a plant family that contains an exclusive, large and still expanding alkaloid group known as the Amaryllidaceae alkaloids, which are characterized by unique skeleton arrangements and a broad spectrum of biological activities [4,5]. Amaryllidaceae plants have been used in folk medicine for their therapeutic and toxic properties [6]. Hippocrates of Kos (460–370 BP), considered the father of modern medicine, recommended the oil of *Narcissus* (Amaryllidaceae) species for the treatment of symptoms that today would be recognized as cancer [7].

**Figure 1:**
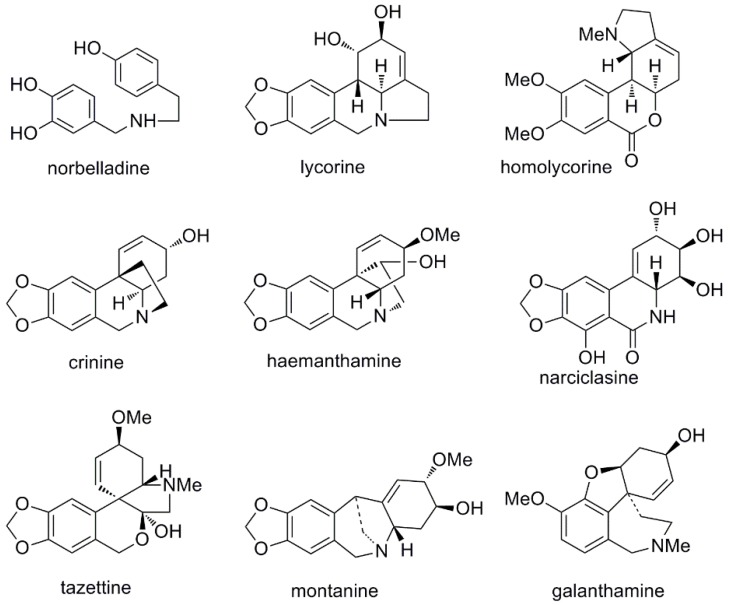
Amaryllidaceae alkaloid types.

The Amaryllidaceae alkaloids are classified mainly into nine skeleton types: norbelladine, lycorine, homolycorine, crinine, haemanthamine, narciclasine, tazettine, montanine and galanthamine (Figure 1) [4]. The most important Amaryllidaceae alkaloid is galanthamine, which was isolated for the first time from *Galanthus woronowii* in the 1950s and was approved for the clinical treatment of mild to moderate Alzheimer’s disease (AD) by the Food and Drug Administration (FDA) at the beginning of this century [8,9]. Alzheimer’s disease (AD), characterized by severe and progressive memory loss, is the most common form of dementia and is becoming increasingly prevalent in people older than 65 years [10,11]. It is estimated that 47 million people live with dementia in the world today, with an economic impact estimated at 818 billion dollars [12]. Known factors involved in the development of AD include a reduced cholinergic neurotransmission level, oxidative stress, amyloid-β-peptide (Aβ) and tau protein aggregation [13].

Acetylcholinesterase (AChE) and butyrylcholinesterase (BuChE) are involved in the hydrolysis of the neurotransmitter acetylcholine (ACh) [14]. Acetylcholinesterase is highly selective for ACh hydrolysis, and BuChE can metabolize different substrates [15]. In the brain of AD patients, AChE activity tends to decrease while that of BuChE increases [16]. Consequently, cholinesterase inhibitors that suppress both AChE and BuChE may provide a better therapeutic response rather than AChE-selective agents [17]. 

Little is known on the chemistry and bioactivity of the South American endemic Amaryllidaceae genus *Rhodophiala*. The *Rhodophiala* species have high ornamental potential due to its attractive red, yellow, white or orange flowers [18,19,20,21,22]. The approximately 40 species described at present occur in Argentina, Bolivia, southern Brazil, Chile and Uruguay [22]. *Rhodophiala* plants have a tunicate bulb of 4–6 cm diameter, which is set 20–30 cm underground, a single umbel holding up to six flowers, each flower being 4–6 cm wide and a flower stem 35 to 50 cm long [18]. 

*Rhodophiala* species are well known ornamental plants. Propagation of the Chilean species was reported [19] as well as phylogenetic [20] and morphological studies [21,22]. The renewed interest on galanthamine sources including the search for additional alkaloids with inhibitory activity towards the enzymes AChE and BuChE has prompted research work on the South American species of this family. Brazilian [23] and Argentinean wild Amaryllidaceae [24] showed high structural diversity in the alkaloid patterns and encouraged work on the Chilean species of this family. The knowledge of the chemical composition and anti-cholinesterase activity of the Chilean species belonging to genus *Rhodophiala* is limited. The comparative studies of their chemical composition are needed to identify the best potential sources of bioactive alkaloids in the South American species. 

The aim of this work was to disclose the potential of Chilean *Rhodophiala* species as inhibitors of the enzymes AChE and BuChE as well as to analyze the alkaloid content and composition of native species by gas chromatography coupled to mass spectrometry (GC-MS) looking for galanthamine sources and searching for other compounds with effect of AChE and BuChE. Molecular docking studies were also carried out to investigate the affinity of the alkaloids identified in the most promising sample at the active sites of AChE and BuChE. 

## 2. Results and Discussion

**Figure 2:**
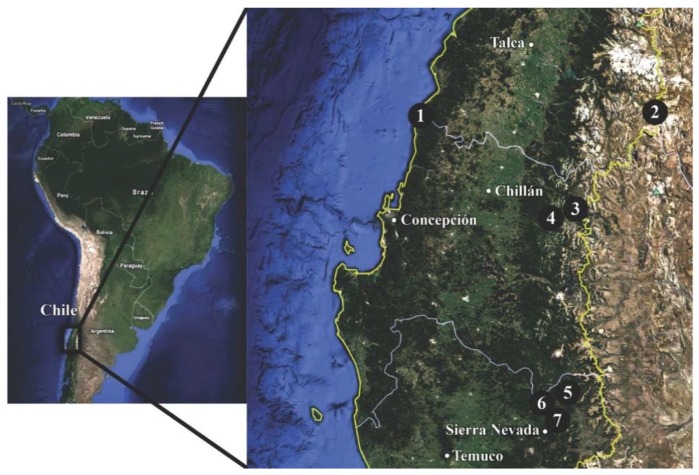
Map of Chile showing the collection sites of the *Rhodophiala* species. 1: Arcos de Calán; 2: Laguna del Maule; 3: Nevado de Chillán; 4: Las Trancas; 5: Volcán Lonquimay; 6: Malalcahuello; 7: Sierra Nevada. Map source: Google Earth.

**Figure 3:**
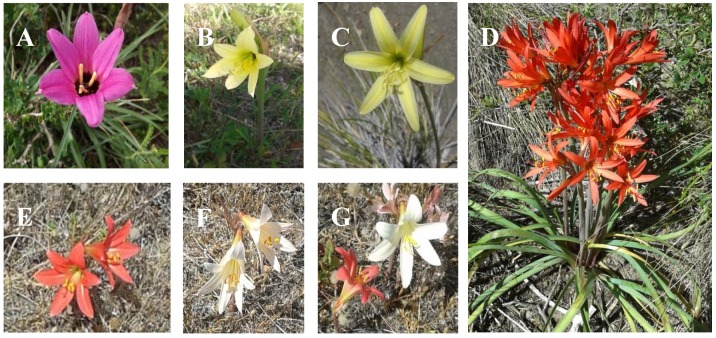
Flowering *Rhodophiala* species from central-southern Chile. (**A**)* R. andicola *(Poepp.) Traub; (**B**) *R. araucana* (Phil.) Traub; (**C**) *R. montana *(Phil.) Traub; (**D**) *R. splendens* (Renjifo) Traub; (**E**) *R. pratensis* (Poepp.) Traub.; (**F**) *R. pratensis* white flowers and (**G**) *R. pratensis* plants with red and white flowers.

Twenty alkaloid extracts from five different Chilean *Rhodophiala* species (Figure 2 and Figure 3) were assessed for inhibitory activity towards the enzymes AChE and BuChE. The alkaloid composition of the extracts was analyzed by GC-MS and the single alkaloid content was quantified and reported as mg GAL/g alkaloid extract (AE). The extracts have been obtained as described in Section 2.2. Briefly, the (fresh) plant material was lyophilized to obtain the dry tissues content (values included in the Table) of the different plant parts investigated. The extraction yields are calculated and reported from the lyophilized plant material.

### 2.1. AChE and BuChE Inhibitory Activities

The crude alkaloid samples from the different Chilean *Rhodophiala* were tested in vitro for AChE and BuChE-inhibitory activity. The percent dry weight of the samples, *w*/*w* extraction yields, and cholinesterase inhibition are summarized in Table 1. Galanthamine was used as a control and presented AChE and BuChE inhibition with IC_50_ values of 0.48 ± 0.07 and 3.70 ± 0.24 μg/mL, respectively. All the alkaloid extracts tested were active against AChE. The highest AChE inhibitory potential was found in bulbs of *R. pratensis* (sample Q) followed by *R. splendens* (sample S) with IC_50_ values of 3.32 ± 0.26 and 3.62 ± 0.02 μg/mL, respectively. Lowest activity was measured for the aerial part of *R. pratensis* (sample N) (IC_50_ value: 102.27 ± 6.61 μg/mL). Nearly 50% of the samples showed some activity against BuChE, with better effect for the bulbs of *R. splendens* (sample S) (IC_50_ 14.37 ± 1.94 μg/mL).

The bulb (I) and leaf (J) extracts of *R. montana* presented moderate activity against both enzymes with better effect of (I) against AChE and (J) towards BuChE. The differences in the chemical profiles of I and J could explain these results. However, the high number of unknown alkaloids in the extracts precludes further discussion. The bulb (Q) and leaf (R) extracts of white flowering *R. pratensis* were active towards AChE, with IC_50_ of 3.32 and 8.39 μg/mL, respectively but with mild to low effect against BuChE (Table 1), reducing the pharmacological interest of this species. The high AChE and BuChE inhibitory activity of *R. splendens* bulb (S) and aerial parts/leaves (T) renders this plant as the most promising species in the search for active molecules for AD therapy (Table 1).

### 2.2. Alkaloid Identification by GC-MS

**Figure 4:**
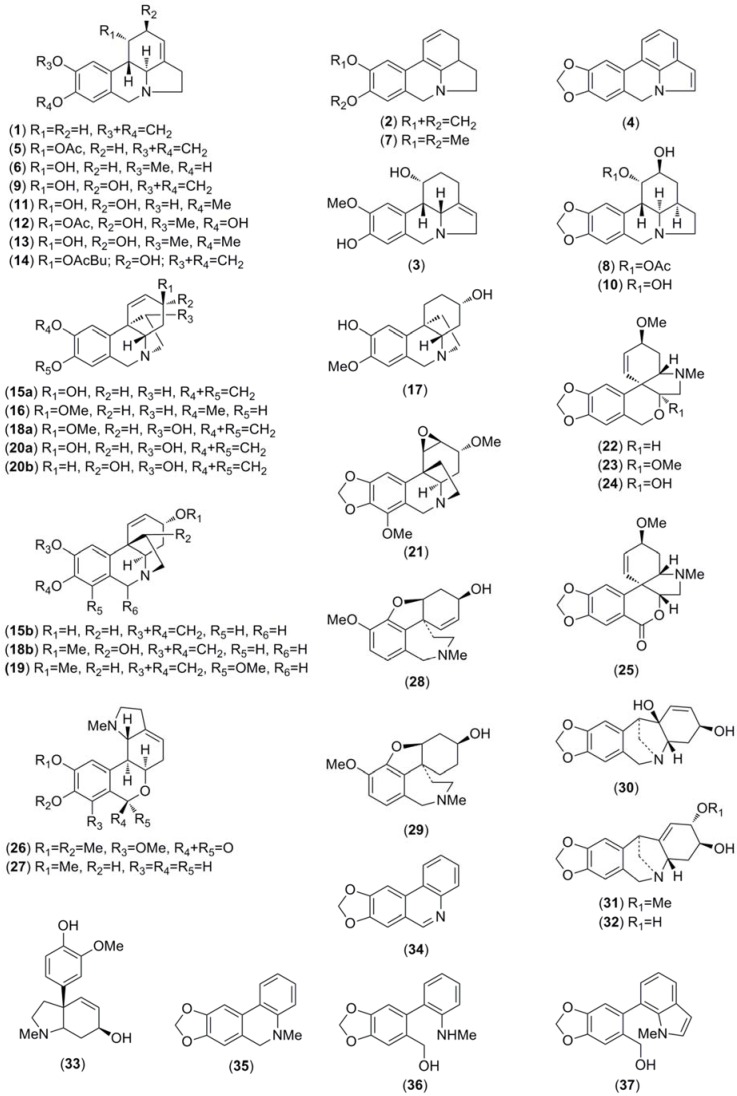
Alkaloids identified in Chilean *Rhodophiala* species by GC-MS.

**Figure 5:**
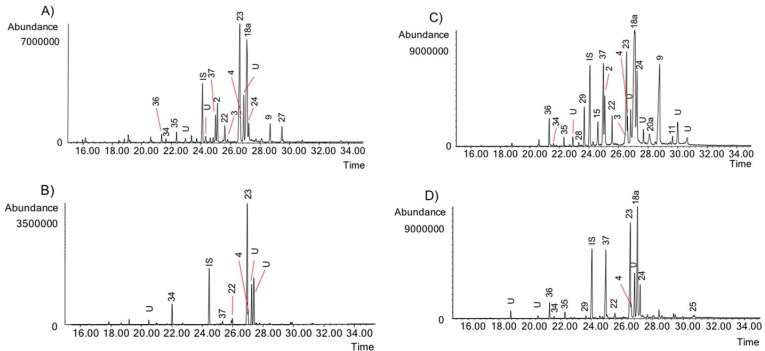
GC chromatograms of the alkaloids from Chilean *Rhodophiala* species. Numbers refer to Table 2. (**A**) Bulbs of *R. andicola* (Sierra Nevada); (**B**) Aerial parts of *R. andicola* (Sierra Nevada); (**C**) Bulbs of *R. andicola* (Nevado de Chillán); (**D**) Aerial parts of *R. andicola* (Nevado de Chillán). IS: internal standard; U: unknown.

**Figure 6:**
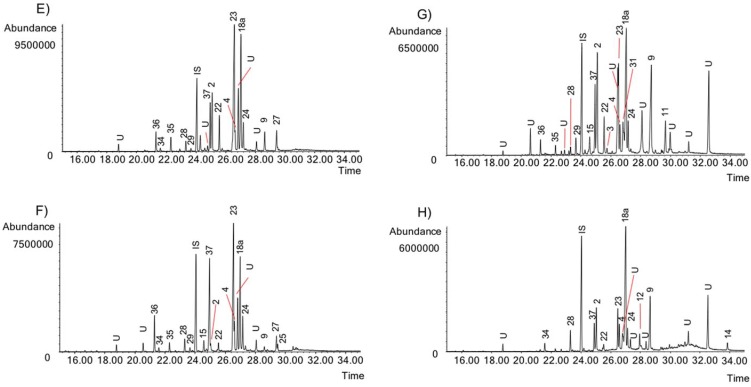
GC chromatograms of the alkaloids from Chilean *Rhodophiala* species. Numbers refer to Table 2. (**E**) Bulbs of *R. andicola* (Volcán Lonquimay); (**F**) Aerial parts of *R. andicola* (Volcán Lonquimay); (**G**) Bulbs of *R. araucana*; (**H**) Aerial parts of *R. araucana*. IS: internal standard; U: unknown.

**Figure 7:**
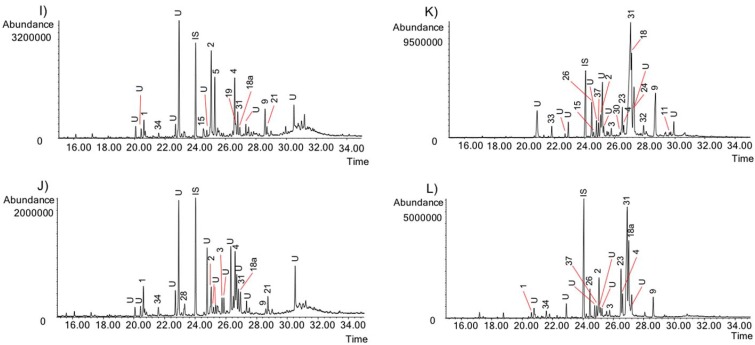
GC chromatograms of the alkaloids from Chilean *Rhodophiala* species. Numbers refer to Table 2. (**I**) Bulbs of *R. montana*; (**J**) Aerial parts of *R. montana*; (**K**) Bulbs of *R. pratensis* (red flowers, without leaves, growing on sand dunes); (**L**) Aerial parts of *R. pratensis* (red flowers, without leaves, growing on sand dunes). IS: internal standard; U: unknown.

**Figure 8:**
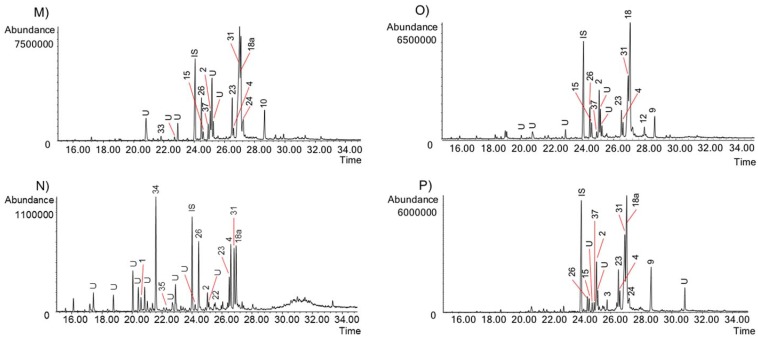
GC chromatograms of the alkaloids from Chilean *Rhodophiala* species. Numbers refer to Table 2. (**M**) Bulbs of *R. pratensis* (red flowers, with leaves); (**N**) Aerial parts of *R. pratensis* (red flowers, with leaves); (**O**) Bulbs of *R. pratensis* (red flowers, without leaves); (**P**) Aerial parts of *R. pratensis* (red flowers, without leaves). IS: internal standard; U: unknown.

**Figure 9:**
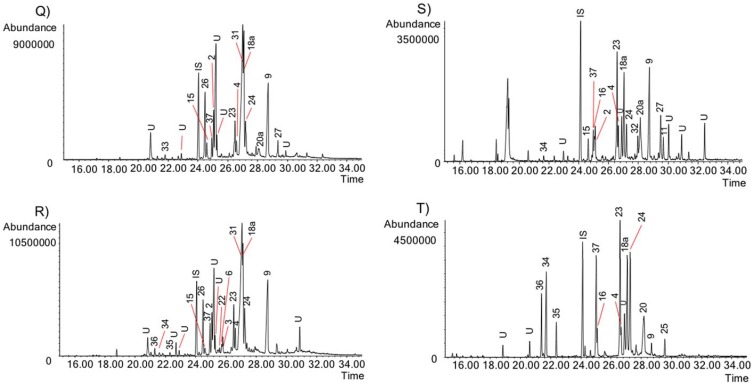
GC chromatograms of the alkaloids from Chilean *Rhodophiala* species. Numbers refer to Table 2. (**Q**) Bulbs of *R. pratensis* (white flowers); (**R**) Aerial parts of *R. pratensis* (white flowers); (**S**) Bulbs of *R. splendens*; (**T**) Aerial parts of *R. splendens*. IS: internal standard; U: unknown.

The activity of the Chilean *Rhodophiala* towards acetylcholinesterase is a consequence of the chemical composition of the extracts. Therefore, the alkaloid composition is a key point to understand the chemical diversity of these plants as a source of potential therapies for AD. The alkaloids occurring in the different extracts were identified by comparing their GC-MS spectra and Kovats Retention Index (RI) values with those of authentic samples. Thirty-seven known alkaloids were identified in these samples (Figure 4). About 50% of them belong to three different alkaloid types, namely: lycorine, haemanthamine and crinine. The others belong to six different alkaloid types: tazettine, homolycorine, galanthamine, montanine, mesembrenone and narciclasine. Two unusual alkaloids known as ismine and galanthindole were also found. The occurrence and quantification of the alkaloids in the samples is summarized in Table 2: (**A**) (aerial parts) and (**B**) (bulbs). The number of alkaloids detected varied among extracts, ranging from 8 in the aerial parts of *R. andicola* collected in Sierra Nevada (B) to 23 in the bulb of *R. pratensis* (K). Forty structures found in these samples could not be identified, suggesting high potential of Chilean *Rhodophiala* species in the search for new alkaloids. The number of unidentified compounds ranged from 3 in aerial parts of *R. andicola* (A, B and D), *R. pratensis* (P) and *R. splendens* (T) to 12 in aerial parts of *R. montana* (J). Representative chromatograms of the samples are shown in Figure 5, Figure 6, Figure 7, Figure 8 and Figure 9.

The highest alkaloid concentration was detected in the aerial parts of *R. andicola* (F) and in the aerial parts of *R. pratensis* (R) (311.1 and 274.1 mg GAL/g AE, respectively). Lowest content was found in the aerial parts of *R. andicola* (B) and in the aerial parts of *R. pratensis* (P) (133.2 and 138.1 mg GAL/g AE, respectively). In 70% of the samples, lycorine-, haemanthamine/crinine- and tazettine-type alkaloids were predominant. Lycorine-type alkaloids were present in all species with higher content in *R. araucana* (G) and *R. montana* bulbs (I) (79.0 and 78.6 mg GAL/g AE, respectively) and lowest values in the aerial parts of *R. andicola* (B) and (D) (8.6 and 7.5 mg GAL/g AE, respectively).

Haemanthamine/crinine-alkaloids occur in all samples except the aerial parts of *R. andicola* (B). However, the higher content was found in the bulbs of *R. andicola* from the same collection place (A) and in the aerial parts of the plant collected at Volcan Lonquimay (F). Compounds from the tazettine-type were not detected in the aerial parts and bulbs of *R. montana* (I and J). From the different Amaryllidaceae alkaloids groups, tazettine-type alkaloids were the main compounds in several samples, occurring in highest concentration in *R. andicola* (E, F) with values of 85.9 and 95.8 mg GAL/g AE of tazettine-type alkaloids in bulbs and aerial parts, respectively. 

Galanthamine-type alkaloids were detected in low quantities in three species, namely *R*. *andicola*, *R. araucana* and *R. montana* (samples C, D, E, F, G, H and J) ranging between 5.1 to 19.0 mg GAL/g AE. Montanine-type alkaloids were present in all species, except *R. andicola*. The highest level of montanine-type compounds was detected in *R. pratensis* (K) (41.7 mg GAL/g AE), which presented three different alkaloids: pancratinine C, montanine and pancracine (5.3, 29.8 and 6.6 mg GAL/g AE, respectively). Mesembrenone-type was the least representative alkaloid-type. It was represented by demethylmesembrenol, detected in low quantities in three different samples of *R. pratensis* (K, M and Q) (7.2, 5.2 and 5.1 mg GAL/g AE, respectively). Narciclasine-type occurs in most samples in a range between 5.1 mg GAL/g AE in bulbs of *R. splendens* (S) to 44.5 and 32.2 mg GAL/g AE in aerial parts of *R. pratensis* with red flowers and leaves (N) and aerial parts of *R. splendens* (T), respectively. All species investigated presented ismine and/or galanthindole alkaloids, except *R. montana*. Forty structures occurring in the extracts could not be identified using the available databases. Three of the unidentified compounds were highly representative among the samples.

The compound with *m*/*z* 252 [M^+^ = 253] (RI 2405.0) occurs in 60% of the samples. The *m*/*z* 109 with [M^+^ = 331] (RI 2557.5), which probably belongs to the homolycorine-type alkaloids, was detected in 40% of the samples and in high amounts in bulbs of *R. pratensis* with white flowers (46.7 mg GAL/g AE). Finally, *m*/*z* 261 with [M^+^ = 345] (RI 2662.6) was detected in 45% of the samples and in high quantity in aerial parts of *R. andicola* collected at Volcan Lonquimay (24.6 mg GAL/g AE). 

The highest content of non-identified alkaloids was detected in the aerial parts of *R. montana* (J) and in the bulbs of *R. pratensis* (red flowers and without leaves) collected in the sand dunes at the sea shore (K) (126.9 and 92.6 mg GAL/g AE, respectively). The lowest content was detected in aerial parts of *R. pratensis* with red flowers and without leaves (P) and in aerial parts of *R. splendens* (T) (20.6 and 21.8 mg GAL/g AE, respectively). 

### 2.3. Molecular Docking

In this study, *R. splendens* was the most active inhibitor of AChE and BuChE. Alkaloid analysis by GC-MS allowed the identification of 17 compounds in the leaf extract of *R. splendens* (T) including two unidentified constituents (Table 2). The 15 alkaloids identified in the extract were evaluated for their theoretical AChE and BuChE inhibitory potential by molecular docking (Table 3 and Table 4). As expected, no alkaloid identified in sample T presented better theoretical AChE inhibitory activity than galanthamine. Molecular simulation of six alkaloids identified in sample T on the 4BDS structure theoretically showed higher enzymatic inhibition against BuChE than galanthamine by 0.80 kcal/mol.

To gain further insight into the molecular docking results, an experiment was carried out to check the AChE and BuChE inhibitory activities of 11-hydroxyvittatine (**20**), lycorine (**9**), 8-O-demethylmaritidine (**16**), hamayne (**20b**), deacetylcantabricine (**17**) and haemanthamine (**18a**) (Table 3). The best AChE and BuChE inhibitory activities were obtained for lycorine (**9**) (IC_50_ 101.70 ± 23.79 μg/mL) and hamayne (**20b**) (IC_50_ 48.40 ± 1.13 μg/mL), respectively. However, their theoretical BuChE inhibition was not supported by the experimental assays. The difference in origin of the BuChE structure used in the molecular docking (human) and experimental assays (equine serum), as well as the inability of these compounds to arrive at the BuChE active site of the enzyme could help to explain the difference between theoretical and practical results.

**Figure 10:**
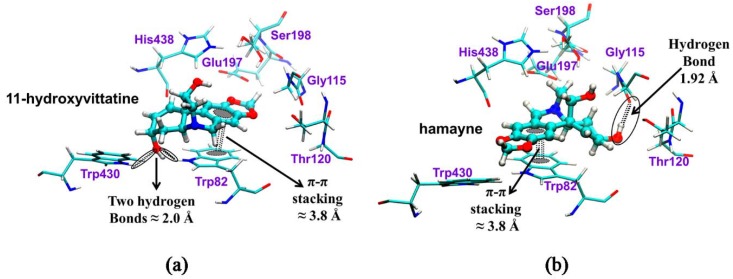
Graphical representations of the binding of (**a**) 11-hydroxivittatine (**20a**) and (**b**) hamayne (**20b**) in the gorge of the active site of *h*BuChE.

Two important regions in the active sites of the hBuChE enzymes have been located: the first corresponding to the catalytic triad composed by the residues His438, Ser198, and Glu325 [25], while the second corresponds to a choline binding site (α-anionic site), composed principally by the residues Trp82 and Phe329 [25]. A graphical representation of molecular binding of 11-hydroxyvittatine (**20a**) and hamayne (**20b**) alkaloids with the hBuChE protein is presented in Figure 10. The alkaloid 11-hydroxyvittatine (**20a**) shows two strong interactions, hydrogen bonds, with the residues Trp82 and Trp430; however, this molecule does not present any interactions close to the catalytic triad His438, Ser198, and Glu325. On the other hand, hamayne (**20b**) shows one hydrogen bond interaction with the residue Gly115, an amino acid located close to the catalytic triad His438, Ser198, and Glu325. In the case of the interactions at the choline binding site (α-anionic site), both alkaloids show the same π–π stacking interaction with the residue Trp82. These molecular interactions suggest that the β-orientation of the hydroxyl group at C-3 in 11-hydroxyvittatine (**20a**) could theoretically increase the BuChE inhibition on the 4BDS structure by 0.49 kcal/mol, compared to the α-orientation of the hydroxyl group at the C-3 position in hamayne (**20b**). However, in the experimental assays, hamayne (**20b**) showed BuChE inhibitory activity (48.40 ± 1.13 μg/mL). It can be hypothesized that the β-orientation of the hydroxyl group at C-3 in 11-hydroxyvittatine (**20a**) probably makes it difficult for the compound to arrive at the catalytic triad in the active site of the BuChE.

Studies on alkaloid composition associated with cholinesterase inhibition and binding-mode prediction have been reported [26,27]. A work on Argentinean Amaryllidaceae [24] reported the composition and acetylcholinesterase inhibition of four wild growing species, including *Rhodophiala mendocina*. Two *R. mendocina* samples collected in different locations presented similar activity towards AChE with IC_50_ values of 2.0 μg/mL but with relevant differences in the qualitative and quantitative alkaloid composition. The sample from the Provincia de San Juan showed high content of haemanthamine/crinamine (31.2%), tazettine (32.9%) and lycorine (13.3%) while the plant collected in the Provincia de Neuquen presented 6.8% haemanthamine/crinamine and 20.4% of lycorine, respectively. Galanthamine was found in both samples with 0.6 and 0.8% for the San Juan and Neuquen plants, respectively. In a report from acetylcholinesterase inhibitory alkaloids from Brazilian Amaryllidaceae [23] the bulbs of *Rhodophiala bifida* (Herb.) Traub were investigated. The activity on AChE was moderate with an IC_50_ value of 8.45 μg/mL, being lower than that from *R. mendocina* [24]. The alkaloid extract of *R. bifida* bulbs contained high amounts of montanine (91.94%). The alkaloid composition of the Chilean *Rhodophiala ananuca* (formerly: *Hippeastrum ananuca*) was described [28,29]. The bulbs contained phenantridine alkaloids, including hippeastidine and epi-homolycorine. 

The alkaloid montanine isolated from *R. bifida* showed activity towards a panel of eight human cancer cell lines. According to [30], montanine at 2.5 μg/mL was more active than doxorubicine on the multi-drug resistant breast cell line NCLADR. Montanine also showed antimicrobial effect with MIC of 5 μg/mL against *S. aureus* ATCC 6538 and *E. coli* ATCC 24922 and 20 μg/mL against *P. aeruginosa* ATCC 27853, respectively [31]. In a screening towards *Trichomonas vaginalis*, dichloromethane and n-butanol extracts from Brazilian *Hippeastrum* species and *Rhodophiala bifida* showed activity against the protozoa [32]. The most active fractions contained the alkaloids lycorine and lycosinine.

In a study on the alkaloids of *Zephyranthes robusta* (Amaryllidaceae), the compounds isolated were evaluated as inhibitors of human cholinesterases [33]. The authors used human erythrocye AChE and serum BuChE. The compounds were tested in a range of 0.5–500 μg/mL and the inhibition was reported as IC_50_ values in μMolar concentration. While the activity of the reference compound galanthamine was similar in both studies, 11-hydroxyvitattine, lycorine and haemanthamine were not active on the human AChE and BuChE. 8-*O*-demethylmaritidine and hamayne were inactive on human BuChE but presented activity on erythrocyte AChE. The differences in the results can be explained by the biological source of the enzymes (human cholinesterases for [33] and electric eel AChE and horse (equine serum) BuChE in this work. For a better comparison of results, the use of enzymes from the same biological source should be recommended. 

In summary, the AChE and BuChE inhibitory activity of the Chilean *Rhodophiala* species investigated led to an interesting source of inhibitors that do not contain the alkaloid galanthamine. Our results suggest that Chilean *Rhodophiala* could be a promising source of new alkaloids with effect towards cholinesterases. The difficulty in finding a species with high activity against AChE and BuChE, the similarity of the AChE and BuChE inhibitory values, the low complexity of the alkaloid profile of aerial parts of *R. splendens*, together with the absence of galanthamine-type alkaloids in this sample, prompted us to further explore the results.

## 3. Materials and Methods

### 3.1. Plant Material

The samples were collected in central-southern Chile and were identified following the reference [19,34]. *Rhodophiala andicola* (Poepp.) Traub, was collected at Sierra Nevada (Región de la Araucanía, 27 January 2016), the slopes of Volcán Lonquimay (Región de la Araucanía, Provincia del Malleco, 19 December 2016) and the slopes of Nevado de Chillán (Región del Bio-Bio, 30 December 2016). *Rhodophiala araucana* (Phil.) Traub was collected at Malalcahuello, Región de la Araucanía (19 December 2016), and *Rhodophiala montana* (Phil.) Traub at the roadside to Laguna del Maule, Región del Maule (2 January 2017). Samples from *Rhodophiala pratensis* (Poepp.) Traub were collected at Arcos de Calán, Región del Maule (12 December 2016) including plants growing on sand dunes and grasslands close to the sea. Plants with red and white flowers were collected separately. According to [34], the plant with red flowers fits the description of *R. pratensis*. *Rhodophiala splendens* (Renjifo) Traub was collected at Las Trancas, Región del Bio-Bio (2 January 2016). The plants were identified by Dr. Patricio Peñailillo, Herbario de la Universidad de Talca. Voucher herbarium specimens have been deposited at the Universidad de Talca as follows: *R. andicola* (N° 4081); *R. araucana* (N° 4083); *R. montana* (N° 4080); *R. pratensis* (N° 4084); *R. pratensis* (white flower) (N° 8085); *R. splendens* (N° 4082). A map with the collection places is shown in Figure 2. Pictures of the species investigated are illustrated in Figure 3. 

### 3.2. Extraction

The freshly collected plant material was cleaned and separated into bulbs and aerial parts, frozen and lyophilized before extraction. The dry weight percentage was determined. The lyophilized plant material was extracted with MeOH under sonication for 10 min (3×) changing the solvent each time. The plant to solvent ratio ranged from 1:10 to 1:60 and was selected according to the volume of plant material for extraction. The combined MeOH solubles were taken to dryness under reduced pressure to afford the crude extracts. The crude extracts were then acidified to pH 3 with diluted H_2_SO_4_ (2%, *v*/*v*) and the neutral material was removed with Et_2_O. The aqueous solutions were basified up to pH 9–10 with NH_4_OH (25%, *v*/*v*) and extracted with EtOAc to provide the alkaloid extracts which were used for all experiments (enzyme inhibition assays and chemical analysis by GC-MS). 

### 3.3. Acetylcholinesterase (AChE) and Butyrylcholinesterase (BuChE) Inhibitory Activity

Cholinesterase inhibitory activities were determined according to [35] with some modifications [36]. Stock solutions with 518U of AchE from *Electrophorus electricus* (Merck, Darmstadt, Germany) and BuChE from equine serum (Merck, Darmstadt, Germany), respectively, were prepared and kept at −20 °C. Acetylthiocholine iodide (ATCI), *S*-butyrylthiocholine iodide (BTCI) and 5,5′-dithiobis (2-nitrobenzoic acid) (DTNB) were obtained from Merck (Darmstadt, Germany). Fifty microliters of AChE or BuChE (both enzymes used at 6.24 U) in phosphate buffer (8 mM K_2_HPO_4_, 2.3 mM NaH_2_PO_4_, 0.15 NaCl, pH 7.5) and 50 μL of the sample dissolved in the same buffer were added to the wells. The plates were incubated for 30 min at room temperature. Then, 100 μL of the substrate solution (0.1 M Na_2_HPO_4_, 0.5 M DTNB, and 0.6 mM ATCI or 0.24 mM BTCI in Millipore water, pH 7.5) was added. After 10 min, the absorbance was read at 405 nm in a Labsystem microplate reader (Thermo Fischer, Waltham, MA, USA). Enzyme activity was calculated as percent compared to a control using buffer without any inhibitor. Galanthamine served as positive control. In a first step, samples were assessed at 10, 100 and 200 μg/mL towards both enzymes. Samples with an IC_50_ > 200 μg/mL were considered inactive. Samples with an IC_50_ < 200 μg/mL were further analyzed to determine the IC_50_ values. The cholinesterase inhibitory data were analyzed with the software Microsoft Office Excel 2010 (Microsoft, Redmond, WA, USA). 

### 3.4. Alkaloids Identification and Quantification

#### 3.4.1. Equipment

The equipment used for the identification and quantification of the alkaloids was a GC-MS 6890N apparatus (Agilent Technologies, Santa Clara, CA, USA) coupled with MSD5975 inert XL operating in the electron ionization (EI) mode at 70 eV. A Sapiens-X5 MS column (30 m × 0.25 mm i.d., film thickness 0.25 μm) was used. The temperature gradient was as follows: 12 min at 100 °C, 100–180 °C at 15 °C/min, 180–300 °C at 5 °C/min and 10 min hold at 300 °C. The injector and detector temperatures were 250 and 280 °C, respectively, and the flow-rate of carrier gas (He) was 1 mL/min. Two mg of each alkaloid extract was dissolved in 1 mL of MeOH:CHCl_3_ (1:1, *v*/*v*) and 1 μL was injected using the splitless mode. Codeine (0.05 mg/mL) was used as an internal standard in all the samples. 

#### 3.4.2. Alkaloids Identification

Amaryllidaceae alkaloids occurring in the samples were identified by comparison of the Rt, fragmentation patterns and data interpretation of the spectra. The database used was built using single alkaloids previously isolated and identified by spectroscopic and spectrometric methods (NMR, UV, CD, IR, MS) in the Natural Products Laboratory, Universidad de Barcelona, the NIST 05 Database and literature data [37,38,39,40,41,42]. 

#### 3.4.3. Alkaloid Quantification

To quantify the single constituents, a calibration curve of galanthamine (10, 20, 40, 60, 80 and 100 μg/mL) was used. The same amount of codeine (0.05 mg/mL) was added to each solution sample as an internal standard. The peak areas were manually obtained considering selected ions for each compound (usually the base peak of their MS, i.e., *m*/*z* at 286 for galanthamine, at 299 for codeine). The ratio between the values obtained for galanthamine and codeine in each solution was plotted against the corresponding concentration of galanthamine to obtain the calibration curve and its equation (y = 0.0224x − 0.2037; R^2^ = 0.9977). All data were standardized to the area of the internal standard (codeine) and the equation obtained for the calibration curve of galanthamine was used to calculate the amount of each alkaloid. Results are expressed as mg GAL, which was finally related to the alkaloid extract weight. As the peak area does not only depend on the corresponding alkaloid concentration but also on the intensity of the mass spectra fragmentation, the quantification is not absolute. However, the methodology is considered suitable to compare the specific alkaloid amount between samples [39,42]. 

### 3.5. Molecular Docking

The molecular docking simulations for the alkaloids identified in *Rhodophiala splendens*, the most promising species found in this study, were performed to investigate the binding mode into the active site of two different enzymes, namely *Torpedo californica* AChE (*Tc*AChE) and *h*BuChE: proteins with PDB codes 1DX6 [43] and 4BDS [44], respectively. The 3D-structures of the alkaloids were drawn using the Chemcraft program [45], and then submitted to a geometrical optimization procedure at PBE0 [46]/6-311+g* [47] level of theory using the Gaussian 09 program [48]. All optimized alkaloids were confirmed as a minimum on the potential energy surface. The docking simulations for the set of optimized ligands were performed using the AutoDock v.4.2 program [49]. AutoDock combines a rapid energy evaluation through pre-calculated grids of affinity potentials with a variety of search algorithms to find suitable binding positions for a ligand on a given macromolecule. To compare the results from the docking simulations, the water molecules, cofactors, and ions were excluded from each X-ray crystallographic structure. Likewise, the polar hydrogen atoms of the enzymes were added, and the non-polar hydrogen atoms were merged. Finally, the enzyme was treated as a rigid body. The grid maps of interaction energy for various atom types with each macromolecule were calculated by the auxiliary program AutoGrid, choosing a grid box with dimensions of 70 × 70 × 70 Å around the active site, which was sufficiently large to include the most important residues of each enzyme. The docking searches for the best orientations of the ligands binding to the active site of each protein were performed using the Lamarckian Genetic Algorithm (LGA) [50]. The LGA protocol applied a population size of 2000 individuals, while 2,500,000 energy evaluations were used for the 50 LGA runs. The best conformations were chosen from the lowest docked energy solutions in the cluster populated by the highest number of conformations. The best docking complex solutions (poses) were analyzed according to the potential intermolecular interactions (ligand/enzyme), such as hydrogen bonding and the cation–π, π–π stacking.

## Figures and Tables

**Table 1:** Percent dry weight, *w*/*w* extraction yields from dry starting material, percent alkaloid extract (from the crude extract), acetyl-(AChE) and butyryl- (BuChE) cholinesterase inhibitory activity of alkaloid-enriched extracts from Chilean *Rhodophiala*.

Scientific Names, Abbreviated Collection Place and Reference Letter	% Dry Weight *^a^	*w*/*w* Extraction Yield *^b^	% Alkaloid Extract *^c^	AChE IC_50_ (μg/mL) *^d^	BuChE IC_50_ (μg/mL) *^d^
**Aerial parts**					
*R. andicola*, SN (B)	15.46	30.63	3.16	18.16 ± 2.94	138.27 ± 6.83
*R. andicola*, NC (D)	18.70	22.10	2.86	12.30 ± 0.74	43.41 ± 2.64
*R. andicola*, VL (F)	20.00	17.50	2.07	74.44 ± 5.53	>200
*R. araucana*, M, (H)	17.70	25.30	3.78	*^e^	*^e^
*R. montana*, LM (J)	21.40	9.40	1.14	33.57 ± 2.16	16.38 ± 0.78
*R. pratensis*, AC, RF, nl, sand dunes (L)	14.84	27.08	1.93	72.59 ± 4.26	>200
*R. pratensis*, AC, RF, L (N)	14.53	10.10	3.72	102.27 ± 6.61	>200
*R. pratensis*, AC, RF, nl (P)	11.68	32.39	2.87	31.97 ± 3.24	>200
*R. pratensis*, AC, WF (R)	10.67	16.47	1.15	8.39 ± 0.27	>200
*R. splendens*, LT, (T)	14.81	44.26	1.53	5.78 ± 0.93	16.26 ± 3.34
**Bulbs**					
*R. andicola*, SN (A)	25.41	7.53	2.15	13.29 ± 1.01	45.76 ± 9.72
*R. andicola*, NC (C)	28.90	6.00	2.23	7.26 ± 0.16	47.38 ± 4.08
*R. andicola*, VL (E)	23.50	7.40	2.37	22.77 ± 1.57	113.24 ± 2.77
*R. araucana*, M, (G)	19.80	7.90	2.73	6.23 ± 0.24	45.71 ± 3.51
*R. montana*, LM, (I)	28.00	3.26	1.50	18.13 ± 0.51	40.05 ± 9.03
*R. pratensis*, AC, RF, nl, sand dunes (K)	18.87	19.73	1.50	11.81 ± 0.17	>200
*R. pratensis*, AC, RF, L (M)	20.98	11.73	2.84	44.23 ± 4.08	>200
*R. pratensis*, AC, RF, nl (O)	18.76	7.36	2.33	47.66 ± 1.78	>200
*R. pratensis*, AC, WF (Q)	19.64	4.84	2.29	3.32 ± 0.26	52.16 ± 0.57
*R. splendens*, LT, (S)	21.35	35.05	1.60	3.62 ± 0.02	14.37 ± 1.94

*^a^ after lyophilization; *^b^ from lyophilized material; *^c^ from the crude extract; *^d^ all IC_50_ were calculated using R^2^ ≥ 0.99; *^e^ insufficient sample; WF: white flowers; RF: red flowers; L: with leaves; nl: no leaves; Collection place: AC: Arcos de Calán, Región del Maule; LM: Laguna del Maule; LT: Las Trancas; M: Malalcahuello, Región de la Araucanía; NC: Nevado de Chillán; SN: Sierra Nevada; VL: volcan Lonquimay.

**Table 2:** (**A**) Identification of alkaloids occurring in the aerial parts of Chilean *Rhodophiala* species by GC-MS. Values are expressed as mg GAL/g AE; (**B**) Identification of alkaloids occurring in the bulbs of Chilean *Rhodophiala* species by GC-MS. Values are expressed as mg GAL/g AE.

**(A)**													
**Alkaloid**	**M^+^**	**BP**	**RI**	**B**	**D**	**F**	**H**	**J**	**L**	**N**	**P**	**R**	**T**
**Lycorine-type**				**8.6**	**7.5**	**25.1**	**46.0**	**46.9**	**35.5**	**37.3**	**35.4**	**60.8**	**13.1**
lycorene (**1**)	255	254	2346.8	-	-	-	-	9.4	5.1	7.2	T	-	-
anhydrolycorine (**2**)	251	250	2543.1	-	T	5.9	11.2	9.6	9.9	8.3	11.8	13.2	-
kirkine (**3**)	253	252	2588.2	-	-	-	-	7.2	5.3	-	6.0	7.2	-
11,12-dehydroanhydrolycorine (**4**)	249	248	2646.4	8.6	7.5	13.5	8.4	15.6	8.1	21.8	7.5	9.4	7.8
1-*O*-acetylcaranine (**5**)	313	226	2653.1	-	-	-	-	-	-	-	-	-	-
norpluviine (**6**)	273	228	2683.6	-	-	-	-	-	-	-	-	6.2	-
assoanine (**7**)	267	266	2708.9	-	T	-	-	-	-	-	-	-	-
3,4-dihydro 1-acetyllycorine (**8**)	331	330	2723.3	-	-	-	-	-	-	-	-	-	-
lycorine (**9**)	287	226	2791.7	-	T	5.7	14.7	5.1	7.1	-	10.1	24.8	5.3
dihydrolycorine (**10**)	289	288	2833.9	-	-	-	-	-	-	-	T	T	-
pseudolycorine (**11**)	289	228	2856.4	-	-	-	-	-	-	-	-	-	-
sternbergine (**12**)	331	228	2838.8	-	-	-	6.4	-	-	-	T	-	-
methylpseudolycorine (**13**)	303	242	2911.2	-	-	-	-	-		-	-	-	-
1-*O*-(3′acetoxybutanoyl)lycorine (**14**)	415	226	3248.8	-	-	-	5.3	-	-	-	-	-	-
**Haemanthamine/crinine type**				**-**	**11.6**	**53.1**	**34.4**	**14.8**	**17.2**	**21.4**	**38.7**	**35.7**	**48.0**
vittatine (**15a**)/crinine (**15b**)	271	271	2512.4	-	T	7.7	-	-	-	-	6.2	5.6	-
8-*O*-demethylmaritidine (**16**)	273	273	2540.0	-	-	-	-	-	-	-	T	-	6.4
deacetylcantabricine (**17**)	275	275	2573.1	-	-	-	-	-	-	-	-	-	T
haemanthamine (**18a**)/crinamine (**18b**)	301	272	2673.4	-	11.6	45.4	34.4	7.7	17.2	21.4	32.5	30.1	25.0
buphanidrine (**19**)	315	315	2748.3	-	-	-	-	-	-	-	-	-	-
11-hydroxyvittatine (**20a**)/hamayne (**20b**)	287	258	2750.5	-	-	-	-	-	-	-	-	-	16.6
undulatine (**21**)	331	331	2892.5	-	-	-	-	7.1	-	-	-	-	-
**Tazettine-type**				**60.7**	**59.1**	**95.8**	**22.3**	**-**	**11.2**	**16.3**	**16.0**	**29.9**	**55.6**
deoxytazettine (**22**)	315	231	2575.6	6.1	5.5	6.7	5.0	-	T	5.2	-	5.2	-
*O*-methyltazettine (**23**)	345	261	2641.1	54.6	36.0	66.2	10.3	-	11.2	11.1	10.1	14.9	30.7
tazettine (**24**)	331	247	2685.1	-	12.5	17.0	7.0	-	-	-	5.9	9.8	24.9
epimacronine (**25**)	329	245	2848.0	-	5.1	5.9	T	-	T	-	-	T	T
**Homolycorine-type**				**-**	**-**	**9.1**	**-**	**-**	**8.6**	**22.4**	**6.5**	**19.5**	**-**
nerinine (**26**)	347	109	2513.5	-	-	-	-	-	8.6	22.4	6.5	19.5	-
8-*O*-demethylhomolycorine (**27**)	301	109	2856.4	-	-	9.1	-	-	-	-	-	-	-
**Galanthamine-type**				**-**	**5.1**	**14.1**	**7.1**	**5.8**	**-**	**-**	**-**	**-**	**-**
galanthamine (**28**)	287	286	2519.9	-	-	8.6	7.1	5.8	-	-	-	-	-
lycoramine (**29**)	289	288	2544.6	-	5.1	5.5	-	-	-	-	-	-	-
**Montanine-type**				**-**	**-**	**-**	**-**	**8.1**	**28.3**	**19.7**	**15.2**	**26.7**	**-**
pancratinine C (**30**)	287	176	2623.5	-		-	-	-	-	-	-	-	-
montanine (**31**)	301	301	2663.1	-	-	-	-	8.1	28.3	19.7	15.2	26.7	-
pancracine (**32**)	287	287	2737.4	-	-	-	-	-	-	-	-	-	-
**Mesembrenone-type**				**-**	**-**	**-**	**-**	**-**	**-**	**-**	**-**	**-**	**-**
demethylmesembrenol (**33**)	275	205	2343.2	-	-	-	-	-	T	-	T	T	-
**Narciclasine-type**				**12.7**	**11.2**	**13.5**	**5.8**	**6.2**	**5.5**	**44.5**	**-**	**10.4**	**32.2**
trisphaeridine (**34**)	223	223	2322.9	12.7	5.1	6.0	5.8	6.2	5.5	39.4	T	5.2	22.7
dihydrobicolorine (**35**)	239	238	2366.1	T	6.1	7.5	T	-	T	5.1	T	5.2	9.5
**Other-type**				**5.2**	**32.9**	**62.1**	**8.5**	**-**	**5.9**	**-**	**5.7**	**17.7**	**34.5**
ismine (**36**)	257	238	2304.6	-	7.6	17.0	T	-	T	-	T	6.0	14.3
galanthindole (**37**)	281	281	2534.8	5.2	25.3	45.1	8.5	-	5.9	-	5.7	11.7	20.2
**Not identified**				**46.0**	**26.8**	**46,1**	**42.0**	**126.9**	**35.9**	**78.5**	**20.6**	**73.4**	**21.8**
unknown (ismine-derivate) *	227	226	2232.2	-	-	-	-	-	-	8.8	-	-	-
unknown (ismine-derivate) *	227	225	2232.2	-	5.6	6.8	5.6	-	-	8.9	-	-	6.2
unknown	269	238	2258.9	-	5.1	6.9	-	-	-	-	-	-	6.4
unknown (mesembrenone-type) *	245	175	2280.8	-	-	-	-		6.0	-	-	10.5	-
unknown	269	268	2285.5	-	-	-	-	-	-	9.7	-	-	-
unknown	253	252	2313.1	5.9	T	-	-	5.9	-	13.7	T	-	-
unknown	251	251	2335.2	-	-	-	-	6.2	-	9.9	-	-	-
unknown (lycorine-type) *	257	256	2379.0	-	-	-	-	-	-	-	-	-	-
unknown	253	252	2405.0	-	-	-	-	8.9	6.2	10.3	-	5.5	-
unknown (lycorine-type) *	269	211	2480.5	-	-	-	T	28.1	-	6.1	T	7.1	-
unknown	271	238	2492.7	-	-	-	-	-	-	-	-	-	-
unknown (homolycorine-type) *	331	109	2557.5	-	-	-	-	-	5.3	5.7	7.0	33.0	-
unknown (crinine/haemanthamine-type) *	329	329	2564.1	-	-	-	-	-	5.3	-	T	7.3	-
unknown	257	225	2579.2	-	-	-	T	-	-	5.4	-	-	-
unknown	299	238	2584.0	-	-	-	-	-	-	-	-	-	-
unknown (crinine/haemanthamine-type) *	315	254	2616.4	-	-	-	-	17.5	6.0	-	5.7	-	-
unknown (tazettine-type) *	315	231	2616.9	-	-	-	-	-	-	-	-	-	-
unknown (lycorine-type) *	329	268	2646.1	-	-	-	-	5.1	-	-	-	-	-
unknown	297	297	2655.7	-	-	-	-	5.4	-	-	-	-	-
unknown (tazettine-type) *	345	261	2662.6	17.9	16.1	24.6	5.6	-	-	-	-	-	9.2
unknown (crinine/haemanthamine-type) *	345	272	2671.8	22.2	-	-	-	-	-	-	-	-	-
unknown (crinine/haemanthamine-type) *	283	283	2692.5	-	-	-	-	6.9	-	-	-	-	-
unknown	303	302	2703.2	-	-	-	-	-	7.1	-	-	-	-
unknown (crinine/haemanthamine-type) *	315	315	2724.2	-		-	-	16.8	-	-	-	-	-
unknown (lycorine-type) *	253	252	2735.3	-	-	-	-	-	-	-	-	-	-
unknown (homolycorine-type) *	345	109	2735.6	-	-	-	-	5.9	-	-	-	-	-
unknown (crinine/haemanthamine-type) *	347	331	2795.5	-	-	-	-	6.5	-	-	-	-	-
unknown (crinine/haemanthamine-type) *	345	331	2795.7	-	-	-	5.6	-	-	-	-	-	-
unknown (crinine/haemanthamine-type) *	347	331	2795.8	-	-	-	-	-	-	-	-	-	-
unknown (lycorine-type) *	251	250	2846.6	-	-	7.8	-	-	-	-	-	-	-
unknown	335	335	2860.6	-	-	-	-	-	-	-	-	-	-
unknown (lycorine-type) *	345	242	2868.8	-	-	-	5.3	-	-	-	-	-	-
unknown	373	372	2881.9	-	-	-	-	-	-	-	-	-	-
unknown (lycorine-type) *	357	356	2942.1	-	-	-	-	-	-	-	7.9	10.0	-
unknown (lycorine-type) *	279	278	3016.0	-	-	-	-	13.7	-	-	-	-	-
unknown (galanthamine-type) *	375	330	3027.5	-	-	-	-	-	-	-	-	-	-
unknown (lycorine-type) *	267	266	3032.9	-	-	-	-	-	-	-	-	-	-
unknown (lycorine-type) *	375	374	3049.2	-	-	-	-	-	-	-	-	-	-
unknown (lycorine-type) *	355	226	3066.6	-	-	-	6.1	-	-	-	-	-	-
unknown (lycorine-type) *	373	226	3161.0	-	-	-	13.8	-	-	-	-	-	-
**Total**				**133.2**	**154.2**	**311.1**	**166.1**	**208.7**	**148.1**	**240.1**	**138.1**	**274.1**	**205.3**
**(B)**														
**Alkaloid**	**M^+^**	**BP**	**RI**	**A**	**C**	**E**	**G**	**I**	**K**	**M**	**O**	**Q**	**S**
**Lycorine-type**				**44.1**	**58.2**	**43.2**	**79.0**	**78.6**	**42.6**	**26.1**	**33.8**	**41.2**	**38.5**
lycorene (**1**)	255	254	2346.8	-	-	-	-	7.6	-	T	-	-	-
anhydrolycorine (**2**)	251	250	2543.1	18.1	15.4	25.0	27.3	24.6	9.4	10.1	13.3	14.9	7.7
kirkine (**3**)	253	252	2588.2	5.0	6.0	-	5.1	T	6.1	-	-	-	T
11,12-dehydroanhydrolycorine (**4**)	249	248	2646.4	11.0	8.6	8.1	8.5	19.0	6.6	6.4	7.3	6.6	8.5
1-*O*-acetylcaranine (**5**)	313	226	2653.1	-	-	-	-	17.4	-	-	-	-	-
norpluviine (**6**)	273	228	2683.6	-	-	-	-	-	-	-	-	-	-
assoanine (**7**)	267	266	2708.9	-	-	-	T	-	-	-	-	-	-
3,4-dihydro 1-acetyllycorine (**8**)	331	330	2723.3	-	-	-	-	-	-	T	-	-	T
lycorine (**9**)	287	226	2791.7	10.0	21.8	10.1	27.5	10.0	15.4	T	7.4	19.7	16.3
dihydrolycorine (**10**)	289	288	2833.9	-	-	-	-	-	T	9.6	T	T	T
pseudolycorine (**11**)	289	228	2856.4	-	6.4	-	10.6	-	5.1	-	-	-	6.0
sternbergine (**12**)	331	228	2838.8	-	-	-	-	-	-	-	5.8	-	-
methylpseudolycorine (**13**)	303	242	2911.2	-	-	-	T	-	-	-	-	-	-
1-*O*-(3′acetoxybutanoyl)lycorine (**14**)	415	226	3248.8	-	-	-	-	-	-	-	-	-	-
**Haemanthamine/crinine type**				**60.0**	**49.5**	**31.7**	**43.7**	**17.4**	**28.6**	**33.8**	**45.5**	**44.9**	**36.9**
vittatine (**15a**)/crinine (**15b**)	271	271	2512.4	-	10.8	-	7.1	T	5.6	6.2	7.3	7.3	6.3
8-*O*-demethylmaritidine (**16**)	273	273	2540.0	-	T	-	-	-	-	-	-	-	6.0
deacetylcantabricine (**17**)	275	275	2573.1	-	-	-	-	-	-	-	-	-	-
haemanthamine (**18a**)/crinamine (**18b**)	301	272	2673.4	60.0	31.7	31.7	36.6	5.8	23.0	27.6	38.2	31.0	16.4
buphanidrine (**19**)	315	315	2748.3	-	-	-	-	5.9	-	-	-	-	-
11-hydroxyvittatine (**20a**)/ hamayne (**20b**)	287	258	2750.5	-	7.00	-	T	-	-	-	-	6.6	8.2
undulatine (**21**)	331	331	2892.5	-	-	-	-	5.7	-	-	-	-	-
**Tazettine-type**				**74.8**	**56.1**	**85.9**	**33.1**	**-**	**21.8**	**18.0**	**8.9**	**17.9**	**21.9**
deoxytazettine (**22**)	315	231	2575.6	8.3	10.7	13.1	9.6	-	-	-	-	-	T
*O*-methyltazettine (**23**)	345	261	2641.1	58.7	23.0	61.7	14.8	-	10.9	12.7	8.9	9.9	14.3
tazettine (**24**)	331	247	2685.1	7.8	22.4	11.1	8.7	-	10.9	5.3	T	8.0	7.6
epimacronine (**25**)	329	245	2848.0	-	T	T	T	-	-	-	-	-	-
**Homolycorine-type**				**9.4**	**-**	**10.6**	**-**	**-**	**13.9**	**14.1**	**10.1**	**28.5**	**9.8**
nerinine (**26**)	347	109	2513.5	-	-	-	-	-	13.9	14.1	10.1	21.1	-
8-*O*-demethylhomolycorine (**27**)	301	109	2856.4	9.4	-	10.6	-	-	-	-	-	7.4	9.8
**Galanthamine-type**				**-**	**19.0**	**11.9**	**12.3**	**-**	**-**	**-**	**-**	**-**	**-**
galanthamine (**28**)	287	286	2519.9	-	5.1	6.8	5.4	T	-	-	-	-	-
lycoramine (**29**)	289	288	2544.6	-	13.9	5.1	6.9	-	-	-	-	-	-
**Montanine-type**				**-**	**-**	**-**	**5.2**	**7.8**	**41.7**	**23.1**	**12.5**	**24.7**	**6.4**
pancratinine C (**30**)	287	176	2623.5	-	-	-	-	T	5.3	T	-	-	-
montanine (**31**)	301	301	2663.1	-	-	-	5.2	7.8	29.8	23.1	12.5	24.7	-
pancracine (**32**)	287	287	2737.4	-	-	-	-	-	6.6	-	-	-	6.4
**Mesembrenone-type**				**-**	**-**	**-**	**-**	**-**	**7.2**	**5.2**	**-**	**5.1**	**-**
demethylmesembrenol (**33**)	275	205	2343.2	-	-	-	-	-	7.2	5.2	T	5.1	-
**Narciclasine-type**				**12.9**	**12.7**	**13.3**	**5.8**	**5.7**	**-**	**-**	**-**	**-**	**5.1**
trisphaeridine (**34**)	223	223	2322.9	5.5	6.3	5.3	T	5.7	T	T	T	T	5.1
dihydrobicolorine (**35**)	239	238	2366.1	7.4	6.4	8.0	5.8	T	T	T	T	T	T
**Other-type**				**19.2**	**35.6**	**28.2**	**21.8**	**-**	**7.9**	**7.8**	**5.3**	**8.0**	**7.3**
ismine (**36**)	257	238	2304.6	6.6	9.9	9.5	6.6	-	T	T	T	T	T
galanthindole (**37**)	281	281	2534.8	12.6	25.7	18.7	15.2	-	7.9	7.8	5.3	8.0	7.3
**Not identified**				**29.9**	**41.4**	**41.2**	**79.8**	**76.8**	**92.6**	**50.5**	**32.0**	**76.0**	**36.0**
unknown (ismine-derivate) *	227	226	2232.2	-	-	-	-	-	-	-	-	-	-
unknown (ismine-derivate) *	227	225	2232.2	-	T	6.2	5.1	-	-	-	T	-	-
unknown	269	238	2258.9	-	T	-	-	-	-	-	-	-	-
unknown (mesembrenone-type) *	245	175	2280.8	-	-	-	-	-	15.3	11.8	6.1	11.4	-
unknown	269	268	2285.5	-	-	-	-	-	-	-	-	-	-
unknown	253	252	2313.1	5.4	-	-	-	6.7	-	-	5.1	-	-
unknown	251	251	2335.2	-	-	-	-	6.2	-	-	-	-	-
unknown (lycorine-type) *	257	256	2379.0	-	-	-	8.0	-	-	-	-	-	-
unknown	253	252	2405.0	-	6.4	-	5.1	6.9	8.3	8.0	6.0	5.4	5.5
unknown (lycorine-type) *	269	211	2480.5	-	-	-	T	34.5	5.3	5.1	-	-	-
unknown	271	238	2492.7	5.7	-	-	-	-		-	-	-	-
unknown (homolycorine-type) *	331	109	2557.5	-	-	-	-	-	19.7	18.5	8.7	46.7	-
unknown (crinine/haemanthamine-type) *	329	329	2564.1		-	-	-	-	6.2	7.1	6.1	7.2	-
unknown	257	225	2579.2	-	-	-	-	-	-	-	-	-	-
unknown	299	238	2584.0	-	-	T	-	-	-	-	-	-	-
unknown (crinine/haemanthamine-type) *	315	254	2616.4	-	-	-	-	5.4	8.6	-	-	-	-
unknown (tazettine-type) *	315	231	2616.9	-	-	5.2	-	-	-	-	-	-	-
unknown (lycorine-type) *	329	268	2646.1	-	-	-	-	-	7.9	-	-	5.3	-
unknown	297	297	2655.7	-	-	-	-	-	-	-	-	-	-
unknown (tazettine-type) *	345	261	2662.6	18.8	11.2	23.0	-	-	-	-	-	-	7.3
unknown (crinine/haemanthamine-type) *	345	272	2671.8	-	-	-	-	-	-	-	-	-	-
unknown (crinine/haemanthamine-type) *	283	283	2692.5	-	-	-	-	-	-	-	-	-	-
unknown	303	302	2703.2	-	-	-	-	-	21.3	-	-	-	-
unknown (crinine/haemanthamine-type) *	315	315	2724.2	-	-	-	-	-	-	-	-	-	-
unknown (lycorine-type) *	253	252	2735.3	-	-	-	17.1	-	-	-	-	-	-
unknown (homolycorine-type) *	345	109	2735.6	-	-	-	-	-	-	-	-	-	-
unknown (crinine/haemanthamine-type) *	347	331	2795.5	-	-	-	-	-	-	-	-	-	-
unknown (crinine/haemanthamine-type) *	345	331	2795.7	-	-	-	-	-	-	-	-	-	-
unknown (crinine/haemanthamine-type) *	347	331	2795.8	-	-	-	-	6.9	-	-	-	-	-
unknown (lycorine-type) *	251	250	2846.6	-	-	6.8	9.9	-	-	-	-	-	-
unknown	335	335	2860.6	-	7.6	-	-	-	-	-	-	-	-
unknown (lycorine-type) *	345	242	2868.8	-	-	-	-	-	-	-	-	-	-
unknown	373	372	2881.9	-	-	-	-	-	-	-	-	-	7.8
unknown (lycorine-type) *	357	356	2942.1	-	-	-	-	-	-	-	-	-	7.0
unknown (lycorine-type) *	279	278	3016.0	-	-	-	-	10.2	-	-	-	-	-
unknown (galanthamine-type) *	375	330	3027.5	-	7.0	-	-	-	-	-	-	-	-
unknown (lycorine-type) *	267	266	3032.9	-	9.2	-	7.3	-	-	-	-	-	-
unknown (lycorine-type) *	375	374	3049.2	-	-	-	-	-	-	-	-	-	8.4
unknown (lycorine-type) *	355	226	3066.6	-	-	-	5.8	-	-	-	-	-	-
unknown (lycorine-type) *	373	226	3161.0	-	-	-	21.5	-	-	-	-	-	-
**Total**				**250.3**	**272.5**	**265.9**	**280.4**	**186.0**	**256.3**	**178.6**	**148.1**	**246.3**	**161.9**

BP: Base Peak; T: Traces; **B**: *R. andicola*, SN; **D**: *R. andicola*, NC; **F**: *R. andicola*, VL; **H**: *R. araucana*; **J**: *R. montana*; **L**: *R. pratensis*, RF, nl, dunes; **N**: *R. pratensis*, AP, RF, L; **P**: *R. pratensis*, RF, nl; **R**: *R. pratensis*, WF; **T**: *R. splendens*; **A**: *R. andicola*, SN; **C**: *R. andicola*, NC; **E**: *R. andicola*, VL; **G**: *R. araucana*; **I**: *R. montana*; **K**: *R. pratensis*, RF, nl, dunes; **M**: *R. pratensis*, RF, L; **O**: *R. pratensis*, RF, nl; **Q**: *R. pratensis*, WF; **S**: *R. splendens*; - : not detected; * proposed structure-type according to the fragmentation pattern.

**Table 3:** AChE and BuChE inhibitory activities of some alkaloids identified in the aerial parts of *R. splendens* (T) and the reference compound galanthamine. Values are expressed as IC_50_ (μg/mL).

Alkaloid	AChE	BuChE
11-hydroxyvittatine (**20a**)	122.17 ± 22.03	>200
lycorine (**9**)	101.70 ± 23.79	>200
8-*O*-demethylmaritidine (**16**)	113.21 ± 8.21	127.87 ± 2.45
hamayne (**20b**)	135.09 ± 15.33	48.40 ± 1.13
deacetylcantabricine (**17**)	>200	>200
haemanthamine (**18a**)	184.68 ± 11.58	>200
galanthamine (**28**)	0.48 ± 0.07	3.70 ± 0.24

**Table 4:** Estimated free energy binding of molecular docking between alkaloids identified in aerial parts of *R. splendens* and cholinesterases (AChE and BuChE). Values are expressed in kcal/mol.

Alkaloid	AChE *^a^	BuChE *^b^
11-hydroxyvittatine (**20a**)	−8.43	−9.03
lycorine (**9**)	−8.82 *^c^	−8.94
8-*O*-demethylmaritidine (**16**)	−8.74 *^d^	−8.93
hamayne **(20b**)	−8.28	−8.54
deacetylcantabricine (**17**)	−7.90	−8.43
haemanthamine (**18a**)	−8.80	−8.34
galanthamine (**28**)	−9.55 *^c^	−8.23 *^c^
epimacronine (**25**)	−9.36 *^c^	−7.63
tazettine (**24**)	−8.66 *^c^	−7.87
*O*-methyltazettine (**23**)	−8.54	−7.87
11,12-dehydroanhydrolycorine (**4**)	−8.41	−7.44
galanthindole (**37**)	−7.81	−7.41
trisphaeridine (**34**)	−7.38	−7.27
dihydrobicolorine (**35**)	−7.33	−7.38
ismine (**36**)	−6.78	−7.08

*^a^ PBD code: 1DX6; *^b^ PBD code: 4BDS; *^c^ Cortes et al., 2015; *^d^ Cortes et al., 2017.
